# Humoral Immune Responses following COVID-19 Vaccinations among Adults in Tanzania

**DOI:** 10.3390/vaccines12010022

**Published:** 2023-12-23

**Authors:** Muhammad Bakari, Said Aboud, Mabula Kasubi, Bruno P. Mmbando, Nyanda Elias Ntinginya, Aifello Sichalwe, Omary S. Ubuguyu, Alex Magesa, Nancy Ladislaus Rutananukwa, Helmut Nyawale, Abisai Kisinda, Medard Beyanga, Pius G. Horumpende, Paulo S. Mhame, Liggle M. Vumilia, Lucy S. Mziray, Reuben Mkala, Elichilia Shao, Abel Makubi, Stephen E. Mshana, Rogath Kishimba

**Affiliations:** 1 School of Medicine, Muhimbili University of Health and Allied Sciences (MUHAS), Dar es Salaam P.O. Box 65001, Tanzania; drbakari@yahoo.com (M.B.); said.aboud@nimr.or.tz (S.A.); 2 National Institute for Medical Research (NIMR), Dar es Salaam P.O. Box 9653, Tanzania; b.mmbando@yahoo.com (B.P.M.); nelias@nimr.mmrc.org (N.E.N.); nancy.rwax@gmail.com (N.L.R.); akisinda@nimr-mmrc.org (A.K.); 3 Muhimbili National Hospital (MNH), Dar es Salaam P.O. Box 65000, Tanzania; mkasubi68@gmail.com; 4 Ministry of Health (MoH), Dodoma P.O. Box 743, Tanzania; chifurumakb@yahoo.co.uk (A.S.); oubuguyu@gmail.com (O.S.U.); alex.magesa@afya.go.tz (A.M.); medbey@hotmail.com (M.B.); piushoru@yahoo.com (P.G.H.); pmhame@yahoo.com (P.S.M.); liggyv@yahoo.com (L.M.V.); lucy.mzirary@afya.go.tz (L.S.M.); abelimakubi@gmail.com (A.M.); rkishimba@moh.go.tz (R.K.); 5 Department of Microbiology and Immunology, Weill Bugando School of Medicine, Catholic University of Health and Allied Sciences (CUHAS), Mwanza P.O. Box 1464, Tanzania; helmut.nyawale@bugando.ac.tz; 6 Benjamin Mkapa Hospital (BMH), Dodoma P.O. Box 11088, Tanzania; rs.mkala13@gmail.com; 7 Kilimanjaro Christian Medical Centre (KCMC), Moshi P.O. Box 3010, Tanzania; elichilia.shao@kcmuco.ac.tz; 8 Faculty of Medicine, Department of Internal Medicine, Kilimanjaro Christian Medical University College (KCMUCo), Moshi P.O. Box 2240, Tanzania; 9 Muhimbili Orthopaedics Institute (MOI), Dar es Salaam P.O. Box 65474, Tanzania

**Keywords:** COVID-19 vaccines, humoral immune response, adults, Tanzania

## Abstract

COVID-19 vaccination remains to be the most important intervention in the fight against the pandemic. The immunity among the vaccinated population and its durability can significantly vary due to various factors. This study investigated the humoral immune responses among individuals who received any of the COVID-19 vaccines approved for use in Tanzania. A total of 1048 randomly selected adults who received COVID-19 vaccines at different time points were enrolled and humoral immune responses (IR) were tested at baseline and three months later (960, 91.6%). The level of SARS-CoV-2 anti-spike/receptor binding domain (RBD) IgG, anti-nucleocapsid IgG, and IgM antibodies were determined using a commercially available chemiluminescent microparticle immunoassay. Descriptive data analysis was performed using STATA version 18 and R. At baseline, serum IgG against anti-spike/RBD was detected in 1010/1048 (96.4%) participants (95%CI: 94.9–97.5) and 98.3% (95%CI: 97.3–99) three months later. The IgG against the SARS-CoV-2 nucleocapsid proteins were detected in 40.8% and 45.3% of participants at baseline and follow-up, respectively. The proportion of seroconverters following vaccination and mean titers of anti-spike/RBD antibodies were significantly more among those who had past SARS-CoV-2 infection than in those with no evidence of past infection, (*p* < 0.001). Only 0.5% of those who had detectable anti-spike/RBD antibodies at baseline were negative after three months of follow-up and 1.5% had breakthrough infections. The majority of participants (99.5%) had detectable anti-spike/RBD antibodies beyond 6 months post-vaccination. The proportion of Tanzanians who mounted humoral IR following COVID-19 vaccination was very high. Seroconversions, as well as the mean titers and durability of humoral IR, were significantly enhanced by exposure to natural SARS-CoV-2 infection. In view of the limited availability of COVID-19 vaccines as well as challenges to completing subsequent doses, booster doses could only be suggested to high-risk groups.

## 1. Introduction

Since the emergency of the SARS-CoV-2 infection that was first recognized in Wuhan, China in December 2019, the entire world has been grappling with an unparalleled health and socio-economic crisis as a consequence. The Coronavirus disease 2019 (COVID-19) led the World Health Organization (WHO) to initially declare the SARS-CoV-2 infection crisis as a Public Health Event of International Concern on 30 January 2020, and later as a pandemic on 12 March 2020. As of 9 August 2023, more than 769,369,823 cases of COVID-19 had been confirmed, including 6,954,336 deaths (https://covid19.who.int/ accessed on 9 August 2023). 

Vaccination has been key to the control of SARS-CoV-2 infection and about 13,492,225,267 doses of COVID-19 vaccines listed by WHO for emergency use have been administered globally by 5 August 2023, which is equivalent to 66.1% of the global population being vaccinated with a complete primary schedule [1]. Tanzania, like other countries in the world, has been adversely affected by COVID-19, with the first patient in the country being confirmed on 16 March 2020. As of 29 July 2022, Tanzania recorded 35,003 cases with an estimated cumulative incidence risk of 62.3/100,000 population. Total deaths recorded were 804, translating into a case fatality rate of 2.3%. Out of the confirmed COVID-19 cases, 3351 were healthcare workers [2]. 

The global scientific response to COVID-19 has been enormous and quite important in terms of understanding the epidemiology of SARS-CoV-2 and providing various tools for diagnosis, therapeutics, and preventive strategies that include vaccines [3]. Although there are currently fairly effective and specific treatments against SARS-CoV-2, it is still strongly believed that the only strategy to combat the virus and control the pandemic is to vaccinate the population [3,4,5]. By 8 August 2023, several COVID-19 vaccine types and/or platforms were listed by the WHO for emergency use and have been widely used since 2020 [6]. Apart from other adopted intervention measures, Tanzania embarked on vaccination from 31 July 2021 with four COVID-19 vaccines, namely, Johnson and Johnson (J&J), Sinopharm, Pfizer, and Moderna [7,8,9,10]. As of 30 June 2023, a cumulative total of 32,905,071 people were fully vaccinated, which is equivalent to 55% of the general population and 100% of adults aged 18 years and above. 

Post-marketing studies have reported evidence that post-vaccine immunogenicity including those in use in Tanzania wanes over time [11]. On the other hand, the magnitude and durability of immune responses have been noted to exhibit individual variability that may be influenced by factors such as age, sex, time since last vaccination, co-morbid conditions (hypertension, diabetes mellitus, obesity) and past SARS-CoV-2 infections [12,13,14,15,16,17]. Consequently, booster doses have been recommended by the WHO [18] and are now being given in various countries. However, of importance is the fact that although the data suggested some variation in levels of protection by vaccine, all the WHO-recommended COVID-19 vaccines have been shown to provide substantial protection against COVID-19 hospitalizations [19], hence calling for continuous emphasis on wider vaccination to all eligible members of the population.

Noticeably, recent studies in Tanzania have reported variable antibody seropositivity rates of SARS-CoV-2 infection, which could also be presumably influenced by natural infection. In two rural villages of Northeastern (Tanga region) Tanzania, the seropositivity rate in children was reported to be 39.7% [20]. Interestingly, a population-based survey in 47 rural villages and 7 peri-urban townships of the same region showed a similar anti-SARS-CoV-2 seroprevalence (39%) among children with mothers having a relatively lower seroprevalence of 29% [21]. However, another study has reported a seroprevalence of 50.4% among adults and children in Mwanza [22]. In Zanzibar, the seropositivity rate has been reported to be 58.9% [23], and this was before the Omicron wave, pointing to the possibility that the majority of Tanzanians have already been exposed to multiple SARS-CoV-2 antigens. 

Though the WHO supports the use of primary and booster dose schedules in the control of the global COVID-19 pandemic [18], data from Tanzania on the magnitude and associated factors of humoral immune responses and their durability post-vaccination remains largely unknown. This information is important for guiding appropriate recommendations towards vaccine strategy in the country. This study aimed to determine the humoral immune responses post-vaccination in recipients of COVID-19 vaccines in mainland Tanzania

## 2. Materials and Methods

### 2.1. Study Design and Study Population

This was a community-based cross-sectional study, whereby individuals from five purposely selected regions were invited to participate in the study by attending specifically selected health facilities. The study involved individuals aged 18 years and above who received a COVID-19 vaccine through the National COVID-19 Vaccination Programme. A list of participants was obtained from the National Electronic COVID-19 vaccination database, which had information on all vaccinated individuals in the country. Permission to access the database was granted by the Permanent Secretary, Ministry of Health. Participants who had received COVID-19 vaccines (namely: Janssen Ad26.COV2.S COVID-19 (Janssen-Cilag International N.V. Turnhoutseweg 30, 2340 Beerse, Belgium), Pfizer-BioNTech BNT162b2 (BioNTech Manufacturing, GmbH An der Goldgrube 12, 55131 Mainz, Germany), Sinopharm BBIBP-CorV (Beijing Institute of Biological Products Co., Ltd. Beijing, China), and Moderna-mRNA-1273 (Moderna, 200 Technology Square, Cambridge, MA, USA) at least two weeks prior to their recruitment into this study were eligible. 

Study participants were randomly selected from five predetermined regions (Dar es Salaam; Dodoma; Mwanza; Kilimanjaro, and Mbeya) using the probability proportion to size (PPS) technique. The selected regions represent the Eastern (Dar es Salaam), Central (Dodoma), Northwestern (Mwanza), Northeastern (Kilimanjaro), and Southern (Mbeya) parts of Tanzania. Selected participants were then invited to the respective study centers (vaccination centers) via phone calls for study enrollment which included administration of a questionnaire and a blood draw to the consenting individuals. They were then invited to come back after three months for a follow-up visit.

### 2.2. Data Collection

A standard questionnaire/data collection tool (Supplementary File S1) was used to collect data on socio-demographic factors, co-morbid conditions, and vaccine-related information. Previous SARS-CoV-2 infection was based on evidence of either a previous positive PCR test result or a history highly suggestive of COVID-19. The comorbidity conditions were established by asking the patient if is suffering from the disease/condition listed in Q21 of the data collection tool. 

### 2.3. Laboratory Procedures

Blood samples from the participating sites included the Muhimbili National Hospital (MNH) in Dar es Salaam; the Mbeya Zonal Referral Hospital (MZRH) in collaboration with NIMR Mbeya Medical Research Centre (MMRC) in Mbeya; the Benjamin Mkapa Hospital (BMH) in Dodoma; the Kilimanjaro Christian Medical Centre (KCMC) in Kilimanjaro; and the Bugando Medical Centre (BMC) in collaboration with the Catholic University of Health and Allied Sciences (CUHAS) in Mwanza and were transported on dry ice packs to the Department of Microbiology and Immunology of the Muhimbili University of Health and Allied Sciences (MUHAS) in Dar es Salaam, within one month of collection. The samples were stored at a −86 °C freezer at MUHAS until the time for batch testing. The blood underwent one freeze-thaw cycle prior to undergoing batch testing (all thawed for the same duration) and was then analyzed at the Central Pathology Laboratory of the MNH, using Abbott Architect SARS-CoV-2 IgM and IgG assays (Abbott Laboratories, Chicago, IL, USA) following manufacturer instructions. The testing algorithm could differentiate serologic activity to SARS-CoV-2 spike S1 subunit, the receptor binding domain (RBD), and the nucleocapsid (N). 

Abbott Architect SARS-CoV-2 IgG (IgG targeting the N-protein: qualitative result only) was used to determine past infection. The Abbott Architect SARS-CoV-2 IgG II Quant (IgG targeting the RBD region of the S-protein) was used for quantitative results to determine IgG levels following vaccination. The SARS-CoV-2 IgM assay detected immunoglobulin class M (IgM) antibodies to the spike protein of SARS-CoV-2 in serum from individuals who may have been acutely infected with SARS-CoV-2. Briefly, the technique involved combining and incubating serum samples, SARS-CoV-2 antigen-coated paramagnetic microparticles, and assay diluent. The IgG and IgM antibodies to SARS-CoV-2 present in the sample bind to the SARS-CoV-2 antigen-coated microparticles. The mixture was then washed, followed by the addition of anti-human IgG/IgM acridinium-labeled conjugate to create a reaction mixture and incubated. Following a wash cycle, Pre-Trigger and Trigger Solutions were added. The resulting chemiluminescent reaction was measured as a relative light unit (RLU). There is a direct relationship between the amount of IgG/IgM antibodies to SARS-CoV-2 in the sample and the RLU detected by the system optics. The relationship was reflected in the calculated index (S/C). The presence or absence of the IgG antibodies to SARS-CoV-2 in the sample was determined by comparing the chemiluminescent RLU in the reaction to the calibrator RLU. Sample to calibrator (S/C) signals of >1.4 and ≥50 were interpreted as reactive to the nucleocapsid and spike assays, respectively. Those samples reactive to the nucleocapsid were considered to be due to natural infection, while those reactive to only the spike protein of the RBD region were considered to be due to vaccination. In addition, a calibrator (S/C) signal ≥ 1.00 was interpreted as reactive to the spike protein in subjects that may have been infected by SARS-CoV-2.

### 2.4. Data Management and Analysis

Data collection tools were developed in the open-source software ‘Open Data Kit v2022.4’ (ODK, https://opendatakit.org/) and collected using tablet computers. All data were synchronized to the main database secured at the Tanga NIMR Centre. A data coordinator ensured the accuracy of data by generating queries that were reconciled by site coordinators. The primary endpoints were the percentage of immune responders post-COVID-19 vaccination using antibody-mediated assays, geometric mean, and antibody titers. 

Data analysis was performed using STATA version 18 and R (https://cran.r-project.org/). Proportions were compared using either Chi-squared or Fisher-exact tests appropriately. Continuous variables were summarized as means with associated standard deviations (SD) or 95% confidence intervals (95%CI) and compared using a *t*-test. Non-normal distributed variables were transformed to normality using logarithm. Univariable and multivariable models (linear and logistic) were used to determine factors associated with the presence and levels of anti-SARS-CoV-2 spike protein. Independent and paired proportions were compared using the chi-squared test (χ2) and McNemar’s test, respectively. Similarly, independent and paired continuous random variables were compared using independent and paired t-tests, respectively. Univariate and multivariate regression models were fitted using generalized estimating equations to determine the association between explanatory and response variables, where the latter was used to control for confounding variables. Only variables with *p*-value < 0.1 or those known to be biologically important to be included were moved to the multivariable models. Quasi-information criteria (QIC) were used to compare models in order to determine which factors were to be retained in the simple models. Results were expressed as coefficients and odds ratio (OR) with associated 95% confidence interval (95%CI) for linear and logistic models, respectively. A *p*-value of 0.05 was regarded as statistically significant.

## 3. Results

### 3.1. Baseline Characteristics (Data Collected on the Enrollment at the First Visit)

A total of 8578 people were available as sampling frame after multiple samples (due to some people not being reachable through the provided mobile phones, relocations, refusal, and wrong address) to obtain the sample size required for each region. Out of 1048 individuals enrolled during the baseline, 960 (91.6%) came back for the follow-up visit. The median age of enrolled participants at baseline was 46 years (IQR: 36–55) and 536 (51.1%) were male. There were slightly more individuals who were older (with a median age of 49 years) as well as more males (58.0%) in Ad26.COV2.S COVID-19 vaccinated participants compared to recipients of other vaccines. The majority of participants were employed (36.4%) and the most (42.2%) had a primary school level of education. The history of being infected with SARS-CoV-2 infection was reported by 78 (7.6%) participants, and out of these, 22 (28.2%) tested positive on PCR. Out of 1,048 enrolled participants, 430 (41.0%) had at least one comorbidity, whereby HIV was the leading condition in 232 (22.1%). A total of 127 (12.1%) had hypertension and 58 (5.5%) had diabetes (Table 1). 

During the follow-up visit, which was implemented three months after the baseline survey, a total of 960 were available for repeat blood sampling and testing. There were no statistically significant differences in social demographic characteristics between participants in the two time points. 

Out of the 1048 enrolled, 414 (39.5%) were vaccinated using the Janssen Ad26.COV2.S COVID-19 vaccine. The proportion of males receiving the Janssen Ad26.COV2.S COVID-19 vaccine was significantly higher when compared to recipients of other vaccines (χ2 = 13.8, *p* = 0.003). The Ad26.COV2.S COVID-19 vaccine was the first vaccine to be introduced, hence the median time since vaccination was 10.4 months (range: 2.5–13.5). Recipients of the Moderna vaccine, which was introduced later, had a median time since vaccination of 3.3 months (range: 2.0–6.7) (Table 2).

### 3.2. Anti-SARS-CoV-2 Spike Protein IgG Antibodies following Vaccination

**Figure 1:**
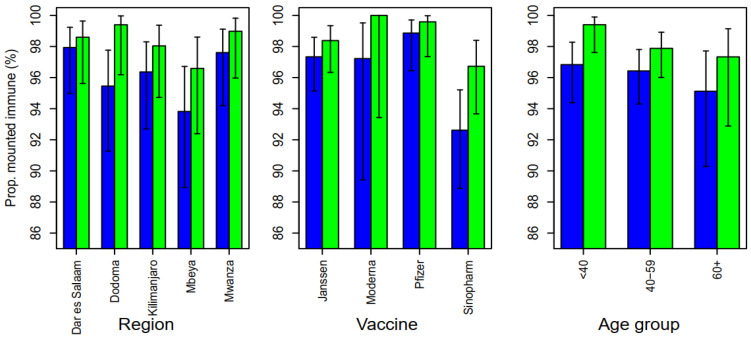
The proportion of participants who had IgG antibodies against the SARS-CoV-2 spike protein following vaccination at baseline (blue) and at follow-up visits (green).

Overall, 96.4% (95%CI: 95.1–97.4) of the participants at baseline seroconverted following vaccination, as determined by the presence of IgG antibodies against SARS-CoV-2 spike proteins. Figure 1 shows the proportion of participants with seroconversion at baseline (blue) and during the follow-up (green) with those who received Pfizer-BioNTech BNT162b2 having the highest proportion of seroconversion (98.9%) at baseline, while those who received the Sinopharm vaccine had the lowest (92.6%) (χ2 = 17.9, *p* < 0.001). 

Mwanza region had the highest proportion of individuals who seroconverted (97.6%), while participants from the Mbeya region had the lowest proportion (93.8%) at baseline, however, the difference was not significantly different (χ2 = 6.39, *p* < 0.172). The seroconversion was found to decrease with increasing age, both at baseline and at follow-up, but the differences were not statistically significant (*p* > 0.05). 

Out of 420 (40.2%) patients who had past COVID-19 infection at baseline, 416 (99.0%) seroconverted, compared to 593/627 (94.6%) who had no past infections (χ2 = 14.4, *p* < 0.001). There was a further increase in the proportion of seroconversion during the follow-up survey (98.3%, 95%CI: 97.3–99.0) when compared to the baseline. Out of 36 individuals who had not seroconverted at baseline, 25 (69%) seroconverted in the follow-up survey; while, out of 16 who were seronegative at follow-up, 5 (31.25%, McNemar’s test = 13.3, *p* < 0.001) were seropositive at baseline. 

### 3.3. SARS-CoV-2 IgM Seropositivity against the Spike Protein

**Figure 2:**
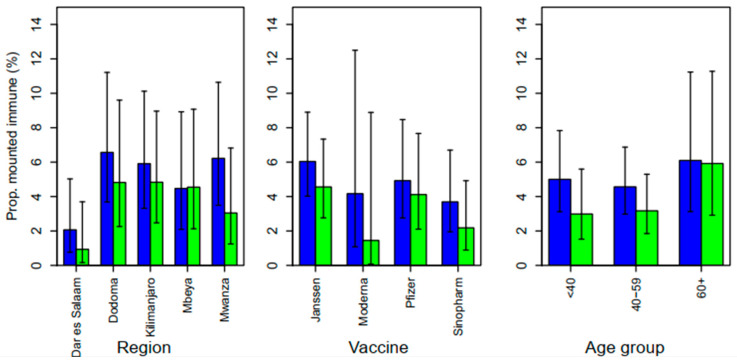
The proportion of participants who mounted immune response and had evidence of acute SARS-CoV-2 infection (IgM) at baseline (blue) and follow-up visit (green).

Overall, 52 (5.0%) of the enrolled participants tested positive for IgM, indicating acute SARS-CoV-2 infection at enrollment, while at follow-up the IgM seropositivity was 3.5%. When compared by vaccine type, the IgM seropositivity ranged from 3.7% for Sinopharm vaccines to 6.0% among those who received the Janssen Ad26.COV2.S COVID-19 vaccine. No statistically significant difference was observed in relation to vaccine type and IgM seropositivity (χ^2^ = 2.136, *p* = 0.545). The proportion of IgM seropositivity was found to range from 2.1% among participants from Dar es Salaam to 6.6% among those from Dodoma. Adults aged 60 years and above (6.1%) had the highest proportion of IgM seropositivity (Figure 2). The proportion of individuals with immune responses during the follow-up (3.5%) was lower than that at the baseline (4.6%) based on paired observations; however, the difference was not significantly different (McNemar’s χ^2^ = 2.63, *p* = 0.105).

Further analysis showed that among the 879 participants who tested negative for IgM at baseline (i.e., no SARS-CoV-2 acute infection) but who developed immune responses following vaccination at the baseline, 13 (1.5%) were IgM seropositive on follow-up (breakthrough infections after vaccination). Of these, 5 out of 229 (2.2%) had received the Pfizer vaccine, 6 out of 340 (1.8%) had received the Janssen & Janssen vaccine, and 2 out of 246 (0.8%) had used the Sinopharm vaccine. 

### 3.4. Anti-SARS-CoV-2 IgG Responses to Nucleocapsid Proteins 

Overall, 40.2% and 45.3% of the participants had a past natural infection as determined by the presence of IgG antibodies against SARS-CoV-2 nucleocapsid proteins at the baseline and follow-up, respectively (McNemar’s test = 7.39, *p* = 0.007). Region-wise, the prevalence of past natural infection at baseline was similar across all regions, with the exception of the slightly higher prevalence in Kilimanjaro, a difference that had marginal significance (χ^2^ = 9.21, *p* =0.056). 

**Figure 3:**
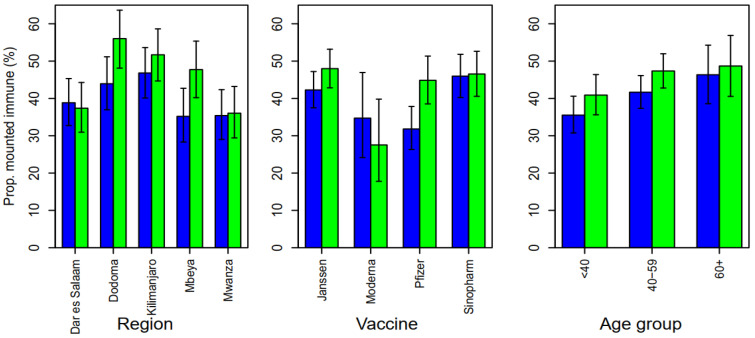
The proportion of participants who mounted IgG antibodies to SARS-CoV-2 nucleocapsid (past infection): Baseline: blue; follow-up: green.

There was a remarkable increase in the proportion of individuals who developed immunity against past infection in the follow-up, which was significant in the Kilimanjaro, (McNemar’s test = 7.68, *p* = 0.006) and Mbeya regions, (McNemar’test = 6.37, *p* = 0.012). The proportion of participants who had a past infection in those who received the Pfizer vaccine (31.8%) was the lowest at baseline, however, there was a remarkable increase in the second round to 44.9%, (McNemar’s test = 11.9, *p* < 0.001). Similarly, the response due to past infection increased by age at the baseline (χ^2^*_trend_* = 6.42, *p* = 0.013) and the follow-up (χ^2^*_trend_* = 3.54, *p* = 0.059) (Figure 3).

### 3.5. Factors Associated with IgG Antibodies to Spike Protein following Vaccination

In multivariate logistic regression analysis, receipt of Sinopharm BBIBP-CorV when compared to receipt of the Janssen Ad26.COV2.S COVID-19 vaccine was associated with the presence of anti-SARS-CoV-2 spike protein antibodies (OR: 0.23, 95%CI: 0.12–046, *p* < 0.001). Similarly, this was the case with past SARS-CoV-2 infection (OR: 13.27, 95%CI: 4.69–37.54, *p* < 0.001). These are shown in Table 3. Furthermore, although not statistically significant, individuals who had a history of suffering from COVID-19 infection were more likely to be seroconverted (OR = 5.24, *p* = 0.114). It is worth noting that, out of 421 individuals who were determined to have an immune response following past natural infection at the baseline, only 36 (8.5%) mentioned having suffered from COVID-19 infection. This proportion was not statistically different from that among participants with no past infection but who reported to have suffered from COVID-19 (42/627, 6.7%), *p* = 0.263.

### 3.6. Anti-SARS-CoV-2 (IgG II) to Spike Protein Levels in COVID-19 Vaccine Recipients at Enrollment 

**Figure 4:**
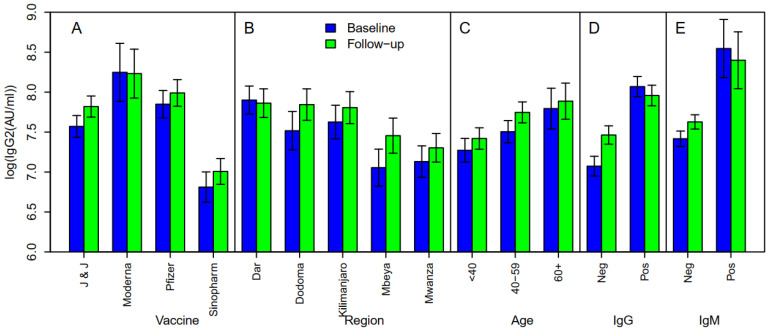
Log geometric mean levels of immune responses following immunization (anti-spike IgG): Baseline: blue; follow-up: green.

The log geometric mean concentrations (GMCs) of anti-IgG SARS-CoV-2 spike protein (AU/mL) varied with the type of vaccine, region, age, past infection status, and acute infection. Furthermore, GMCs were higher during the follow-up visit than at the baseline for the majority of the variable categories presented in Figure 4. The GMC levels of those who received Moderna and Pfizer vaccines were higher, while that of recipients of the Sinopharm vaccine was lower when compared to those who received the Janssen vaccine.

Participants from Dar es Salaam had the highest GMC of IgG anti-SARS-CoV-2 spike protein antibodies, while lower levels were observed among participants from Mbeya. The GMCs of anti-IgG SARS-CoV-2 spike protein were found to increase with age, with the highest GMCs observed in those 60 years and above (*p* < 0.001). 

Furthermore, it was observed that participants (baseline) who had past natural infection had significantly higher GMCs of anti-IgG SARS-CoV-2 spike protein antibody (8.07 ± 0.13 AU/mL) than those without past natural infection (7.07 ± 0.12 AU/mL, *p* < 0.001). The same results were observed during follow-up (8.12 ± 0.10 AU/mL vs. 7.28 ± 0.12 AU/mL, *p* < 0.001). 

**Figure 5:**
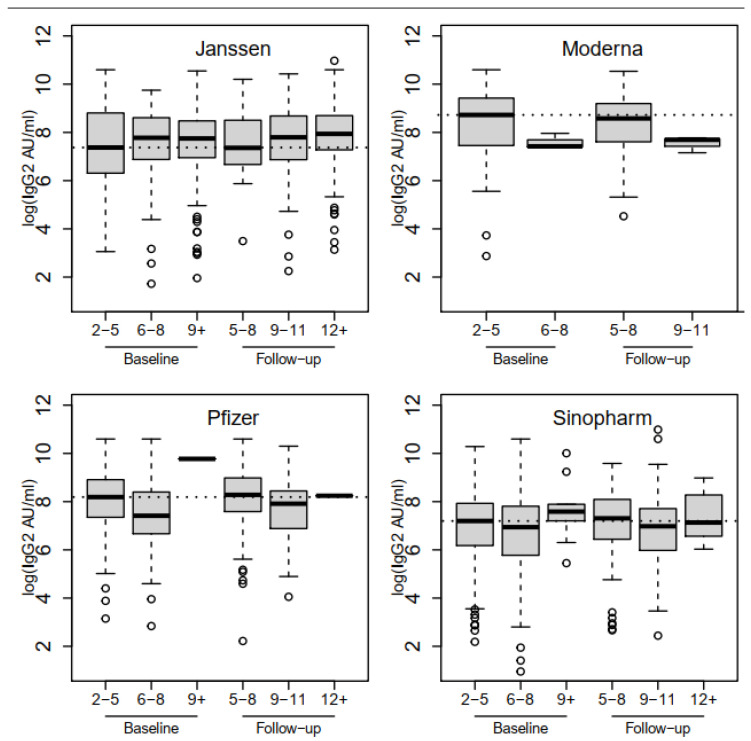
Distribution of levels (log scale) of immune titers (IgG levels) following vaccination by vaccine type and time since COVID-19 vaccination.

When analyzed by vaccine type, those who received the Janssen Ad26.COV2.S COVID-19 vaccine had levels of anti-SARS-CoV-2 IgG antibodies that increased significantly (0.19, 95%CI: 0.07–0.32, *p* = 0.002) over time, with high levels still being observed at 12 months since vaccination. The levels of those who received the Moderna, Pfizer, and Sinopharm vaccines decreased with time when compared to those among vaccines who received the Janssen vaccine, with a similar pattern between the baseline and follow-up periods (Figure 5). It was interesting to note that, although the antibody titers among individuals who received the Janssen Ad26.COV2.S COVID-19 vaccine were slightly lower than that of other vaccines with reference to recipients of Sinopharm BBIBP-CorV vaccine (−0.64 (95%CI: −1.61–0.32), *p* = 0.192), the levels among the recipients of the Janssen Ad26.COV2.S COVID-19 vaccine increased with the duration since vaccination (0.19 (95%CI: 0.07–0.32), *p* = 0.002).

In multivariate analysis, factors found to be associated with higher GMCs of SARS-CoV-2 anti-spike protein antibodies were vaccine type, sex, survey round, and past and acute infection status. Overall, the follow-up visit was found to be significantly associated with significantly high GMCs, 0.16 (95%CI: 0.008–0.23, *p* < 0.001) compared to baseline. Table 4 shows that levels were significantly high in recipients of Moderna and Pfizer vaccines, but were low in recipients of Sinopharm vaccine when participants of the Janssen Ad26.COV2.S COVID-19 vaccine were regarded as a reference. Furthermore, past infection (0.91, *p* < 0.001) and acute infection (0.69, *p* < 0.001) were associated with a significant increase in the levels of SARS-CoV-2 anti-spike protein IgG antibodies (Table 4).

## 4. Discussion

The study findings show that COVID-19 vaccines administered in Tanzania led to seroconversion in the majority of individuals, regardless of participants’ age though with a slight increase in the proportion of seroconverters after three months of follow-up. It was further observed that only 0.5% of participants lost their humoral immune response after three months of follow-up with the majority having detectable antibodies beyond 6 months post-vaccination (Figure 5). The breakthrough infections were observed in 1.5% of participants with more cases from those who received the Pfizer-BioNTech BNT162b2 vaccine. Furthermore, the study findings have shown that previous/past SARS-CoV-2 infection significantly contributed to seroconversion and the corresponding levels of humoral immune responses among COVID-19-vaccinated individuals (Table 4 and Figure 4). Our study findings have shown a seroconversion proportion that is greater than 90% across the four vaccines (Janssen Ad26.COV2.S COVID-19, Pfizer-BioNTech BNT162b2, Sinopharm BBIBP-CorV, and Moderna COVID-19 (mRNA-1273)) both at baseline and follow-up, with the highest (99%) and the lowest (94%) proportions being reported among vaccines who received the Pfizer-BioNTech BNT162b2 and Sinopharm BBIBP-CorV, respectively (Figure 1). Noticeably, the majority of enhanced humoral responses have been observed among participants who received mRNA-based vaccines, as reported earlier [24]. However, there are also few reports on good immune responses following receipt of an adenovirus-based vaccine [25]. 

Despite the majority of studies showing neutralizing antibody titers being maintained for about six months, other studies have found durable immunity for up to 10–12 months after infections [26,27]. In the current study, we have observed that there was no difference in immunity at two and 12 months post-vaccination among those who received the Janssen Ad26.COV2.S COVID-19 vaccine with the majority of those who received the Janssen Ad26.COV2.S COVID-19 vaccine showing detectable antibodies beyond 12 months post-vaccination. Furthermore, previous studies have shown a negative correlation between age and antiSARS-CoV-2 RBD IgG levels [15,17,28]; however, in the current study, those with 60 years of age and above had a significantly higher geometric mean of antibody titers than those below 60 years (Table 4). The 60 years and above group could have been heavily exposed to natural SARS-CoV-2 infection and the natural immunity mounted during priming could have been augmented/boosted by the COVID-19 vaccination. Two previous studies have also observed higher SARS-CoV-2 antibody levels in older people than in younger age groups [29,30]. 

It is well documented that higher IgG levels against spike proteins have been found to be associated with immunity to SARS-CoV2 infection [24]. Antibody responses after SARS-CoV-2 infection and/or COVID-19 vaccination [31,32,33] are critical parts of antiviral immunity. Such mounted Infection-neutralizing antibody levels are reported to be highly predictive of immune protection from future symptomatic SARS-CoV-2 infection [34] including emerging virus variants that have evolved to escape previous antibodies and thus important for public health policy strategies. Therefore, exposure to multiple SARS-CoV-2 spike antigens, whether following vaccination and/or natural infection has been reported to enhance hybrid immunity (previous infection SARS-CoV-2 variants plus vaccination) with subsequent neutralization of circulating variants of concerns [35]. This observation is further confirmed in the current study whereby a high hybrid immune response was mounted with natural infection observed in the current study being comparable to seroprevalence studies that have been recently reported in selected parts of Tanzania [20,22,23]. This points to the possibility of the majority of Tanzanians being exposed to multiple SARS-CoV-2 antigens. Noticeably, the hybrid immunity in our findings is reportedly more pronounced among participants with mRNA-based vaccination following natural infection. 

It would, therefore, appear that in settings with adherence challenges to complete the subsequent doses as well as in areas with inequitable access to vaccines, arguably giving a single dose is useful, bearing in mind a background of natural infection confers the desired immunity. This is further supported by a recent study by Powell et al. in England among adolescents, which showed that hybrid immunity provides sustained immunity as compared to vaccination alone, which provides low-to-moderate protection, with waning protection after each dose especially against symptomatic Omicron infection [36]. This finding could arguably preclude the requirement for additional booster vaccine doses, especially in low-risk groups.

In the current study where all participants were enrolled more than 2 months after vaccination, 5.5% of the enrolled participants were IgM seropositive at the time of enrollment indicating acute SARS-CoV-2 infection before immunity was mounted due to either underlying factors or COVID-19 vaccine breakthrough infections. Furthermore, we observed that 1.5% of those who received vaccines and had mounted immunity were acutely infected within three months of follow-up. These data confirm the possibility of vaccine breakthrough infections which are increasingly reported following the emergence of new variants [37]. These observations could explain the aforementioned proportion of infections post-vaccination in our study as the evaluation period encompasses the time when the Omicron variant was prominent in Tanzania. Individuals with COVID-19 vaccine breakthrough infections have a small potential to spread SARS-CoV-2 to other people compared to unvaccinated individuals and are less likely to have severe COVID-19 symptoms and diseases compared to the latter [37]. A similar observation has been noticed in our study, hence further underscoring the need for continuous vaccination across the country.

Limitations: This community-based study representing different geographic locations in the country has the following limitations: The study included only populations who opted for COVID-19 vaccination; therefore, results such as past infection need to be interpreted carefully because of the potential selection bias. Furthermore, there is a possibility of bias toward people with certain comorbidities such as HIV who might be expected to benefit more from the vaccine and this might have affected the parameter estimates. 

## 5. Conclusions

The study findings show that most adult Tanzanians who received COVID-19 vaccines mounted a humoral immune response against SARS-CoV-2 infection irrespective of the vaccine types and the response was significantly enhanced by natural infection. We also observed that in the majority of the participants, the immune response was still detectable at three months of follow-up and beyond six months post-vaccination. In view of the limited availability of COVID-19 vaccines as well as the challenges to complete subsequent doses, booster doses should be emphasized to be administered to high-risk groups. 

## Figures and Tables

**Table 1:** Social demographic and clinical characteristics of study participants (N = 1048) at enrollment by region.

Characteristics	Region					
	Dar es Salaam	Dodoma	Kilimanjaro	Mbeya	Mwanza	Overall
Sample Size (n)	242	198	220	179	209	1048
Mean age (years), Median (IQR)	45 (39–53)	46 (37–55)	50 (41–59)	46 (35–57)	38 (30–52)	46 (36–55)
Sex (male, %)	99 (40.9)	111 (56.0)	114 (51.8)	107 (59.8)	105 (50.2)	536 (51.1)
Education level, n (%)						
No formal education	17 (7.0)	3 (1.5)	8 (3.6)	4 (2.3)	7 (3.3)	39 (3.7)
Primary school	129 (53.3)	62 (31.3)	113 (51.3)	58 (32.4)	80 (38.2)	442 (42.1)
Secondary school	62 (25.6)	40 (20.2)	48 (21.8)	33(24.0)	62 (29.7)	255 (24.4)
College	13 (5.4)	32 16.2)	26 (11.8)	32 (17.9)	29 (13.4)	132 (12.6)
University and above	21 (8.7)	61 (30.8)	25 (11.4)	42 (23.5)	31 (14.8)	180 (17.2)
Occupation, n (%)						
Formerly employed	64 (26.5)	94 (47.5)	63 (28.6)	81 (45.3)	80 (38.3)	382 (36.4)
* Farmer/Peasant (crops)	12 (5.0)	28 (14.1)	66 (30.0)	46 (25.7)	21 (10.0)	173 (16.5)
** Livestock keeping	3 (1.2)	2 (1)	62 (28.2)	1 (0.6)	0 (0)	68 (6.5)
Business	107 (42.2)	55 (27.8)	5 (2.3)	43 (24.0)	62 (30.0)	272 (26.0)
Others	56 (23.1)	19 (10.0)	24 (10.9)	8 (4.5)	46 (22.0)	153 (15.0)
History of COVID-19, n (%)	1 (0.4)	54 (27.3)	15 (6.8)	2 (1.1)	6 (2.9)	78 (7.4)
Diagnosed with COVID-19, n (%)	1 (100)	10 (18.5)	4 (26.7)	2 (100)	5 (83.3)	22 (28.2)
Any comorbidity, n (%)	176 (72.7)	77 (38.9)	60 (27.3)	45 (25.1)	72 (34.4)	430 (40.0)
Hypertension	28 (11.6)	38 (19.2)	17 (7.7)	23 (12.8)	21(10.0)	127 (12.1)
Diabetes	13 (5.4)	14 (7.1)	10 (4.5)	10 (5.6)	11 (5.3)	58 (5.5)
HIV	147 (60.7)	19 (9.6)	20 (9.0)	11 (6.1)	35 (16.7)	232 (22.1)
Median time since vaccination, months (Range)	5 (3.3–7.1)	7.5 (5.8–10.8)	6.8 (5.2–10.3)	8.4 (6.6–10.5)	6.9 (5.6–9.1)	6.9 (5.0–10.1)

* Farmers (peasant): Those engaging in farming activities on a small scale. ** Livestock keeping: Those engaging in keeping poultry and domestic animals (cows, goats, etc.).

**Table 2:** Social demographic and clinical characteristics of study participants (N = 1048) at enrollment by vaccine type.

Characteristics	Vaccine Type				
	Jensen & Jensen	Moderna	Pfizer	Sinopharm	Overall
Sample Size (n, %)	414 (39.5)	72 (6.9)	264 (25.2)	298 (28.4)	1048
Mean age (years), Median (IQR)	49 (39–58)	43.5 (32–51)	43 (33–52)	43 (34–54)	46 (36–55)
Sex (male, %)	240 (58.0)	28 (39.0)	128 (48.5)	140 (47.0)	536 (51.1)
Education level, n (%)					
No formal education	10 (2.4)	6 (8.3)	9 (3.4)	14 (4.7)	39 (3.7)
Primary school	145 (35.0)	45 (62.5)	113 (42.8)	139 (46.6)	442 (42.2)
Secondary school	95 (22.9)	15 (20.8)	65 (24.6)	80 (26.9)	24.4 (24.4)
College	60 (14.5)	3 (4.2)	41 (15.5)	28 (9.4)	132 (12.6)
University and above	104 (25.1)	3 (4.2)	36 (13.6)	37 (12.4)	180 (17.2)
Occupation, n (%)					
Formerly employed	185 (44.7)	17 (23.6)	94 (35.6)	86 (28.9)	382 (36.4)
Farmer/Peasant (crops)	69 (16.7)	7 (9.7)	45 (17.0)	52 (17.4)	173 (16.5)
Livestock keeping	25 (6.0)	3 (4.2)	24 (9.0)	16 (5.4)	68 (6.5)
Business	71 (17.1)	32 (44.4)	67 (25.4)	102 (34.2)	272 (25.9)
Others	64 (15.5)	13 (18.1)	34 (12.9)	42 (14.1)	153 (14.6)
History of COVID-19, n (%)	35 (8.4)	2 (2.8)	20 (7.6)	21 (7.0)	78 (7.4)
Diagnosed with COVID-19, n (%)	11 (31.4)	1 (50.0)	5 (25.0)	5 (23.8)	22 (28.2)
Any comorbidity, n (%)	151 (36.5)	51 (70.8)	100 (37.9)	128 (42.9)	430 (41.0)
Hypertension	70 (16.9)	3 (4.2)	18 (7.1)	38 (12.7)	127 (12.4)
Diabetes	36 (8.7)	2 (2.8)	9 (3.4)	11 (3.7)	58 (5.5)
HIV	32 (8.2)	47 (56.6)	60 (23.5)	83 (27.8)	232 (22.1)
Median time since vaccination, months (Range)	10.4 (9.3–11.3)	3.3 (2.8–3.4)	5.6 (4.4–6.4)	6.3 (5.0–7.4)	6.9 (5.0–10.1)

**Table 3:** Univariable and multivariable analyses showing factors associated with immune response following immunization (spike IgG antibodies, qualitative).

	Univariable			Multivariable		
Variable	OR	95%CI	*p*-Value	OR	95%CI	*p*-Value
Age group						
<40	1			1		
40–59	0.88	0.42–1.86	0.742	0.54	0.27–1.09	0.085
60+	0.64	0.26–1.59	0.335	0.43	0.19–1.00	0.050
Vaccine:—J&J	1			1		
Moderna	0.96	0.21–4.41	0.956	0.75	0.16–3.61	0.724
Pfizer	2.38	0.66–8.61	<0.001	2.54	0.83–7.71	0.099
Sinopharm	0.34	0.16–0.72	0.005	0.23	0.12–0.46	<0.001
Comorbidity:—No	1					
Diabetes	0.67	0.2–2.26	0.521			
Hypertension	1.63	0.5–5.39	0.420			
HIV	1.27	0.55–2.92	0.573			
Suffer COVID-19						
No	1			1		
Yes	3.06	0.41–22.58	0.273	5.24	0.67–40.99	0.114
Past SARS-CoV-2 infection						
No	1			1		
Yes	5.96	2.1–16.93	0.001	13.27	4.69–37.54	<0.001
Time since vaccination (months)	1.04	0.92–1.16	0.550			
Survey round						
Baseline	1			1		
Follow-up	2.21	1.23–3.99	0.008	2.24	1.22–4.13	0.010
Region						
Dar es Salaam	1			1		
Dodoma	0.44	0.15–1.34	0.151	0.30	0.11–0.82	0.019
Kilimanjaro	0.56	0.18–1.74	0.314	0.30	0.11–0.79	0.015
Mbeya	0.32	0.11–0.94	0.038	0.22	0.09–0.57	0.002
Mwanza	0.86	0.25–3.02	0.815	0.68	0.24–1.98	0.485

**Table 4:** Factors associated with levels of immune response following vaccination.

	Univariate		Multivariate	
Variable	Coefficient (95%CI)	*p*-Value	Coefficient (95%CI)	*p*-Value
Age group (yrs.)				
40–59	0.27 (0.09–0.45)	0.003	0.09 (−0.06–0.25)	0.246
60+	0.51 (0.27–0.75)	<0.001	0.4 (0.19–0.61)	<0.001
Sex (Female)	0.15 (−0.01–0.32)	0.063	0.17 (0.03–0.3)	0.018
Time since vaccination (months)	−0.012 (−0.04–0.02)	0.419		
Survey round—Follow-up	0.19 (0.11–0.27)	<0.001	0.16 (0.08–0.23)	<0.001
Type of vaccine				
Moderna	0.62 (0.30–0.93)	<0.001	0.59 (0.29–0.88)	<0.001
Pfizer	0.22 (0.03–0.42)	0.025	0.31 (0.14–0.49)	<0.001
Sinopharm	−0.79 (-0.98–−0.61)	<0.001	−0.84 (−1.01–−0.67)	<0.001
Region				
Dodoma	−0.23 (−0.48–0.01)	0.065	−0.53 (−0.76–−0.29)	<0.001
Kilimanjaro	−0.16 (−0.4–0.08)	0.180	−0.47 (−0.69–−0.26)	<0.001
Mbeya	−0.63 (−0.88–−0.38)	<0.001	−0.67 (−0.89–−0.44)	<0.001
Mwanza	−0.68 (−0.93–−0.44)	<0.001	−0.75 (−0.96–−0.53)	<0.001
Suffered COVID-19	0.71 (0.35–1.08)	<0.001	0.73 (0.45–1.01)	<0.001
Immunity against past infection (IgG): Yes	0.91 (0.80–1.01)	<0.001	0.91 (0.8–1.01)	<0.001
Immunity against acute infection (IgM): Yes	0.80 (0.50–1.10)	<0.001	0.69 (0.42–0.96)	<0.001
Comorbidity—None				
Diabetes	0.37 (−0.016–0.72)	0.041		
Hypertension	0.26 (0.01–0.51)	0.038		
HIV	0.16 (−0.03–0.36)	0.106		
Asthma	0.21 (−0.30–0.72)	0.421		

## Data Availability

All data have been included in the article.

## Supplementary Materials

The following supporting information can be downloaded at: https://www.mdpi.com/article/10.3390/vaccines12010022/s1, Supplementary File S1: Data collection tool.
