# Supplementary material for: Humoral Immune Responses following COVID-19 Vaccinations among Adults in Tanzania
Source: Vaccines (Basel). 2023 Dec 23;12(1):22. doi: 10.3390/vaccines12010022 (PMC10819524; doi:10.3390/vaccines12010022)
Supplement: Supplementary file 1 [file vaccines-12-00022-s001.zip › vaccines-2748610-supplementary.pdf]

MINISTRY OF HEALTH, COMMUNITY DEVELOPMENT, GENDER, ELDERLY AND CHILDREN (MOHCDGEC) AND MUHIMBILI UNIVERSITY OF HEALTH AND ALLIED SCIENCES (MUHAS)

DATA COLLECTION TOOL: ENGLISH VERSION

STUDY TITLE: IMMUNOGENICITY FOLLOWING ADMINISTRATION OF COVID-19 VACCINES IN TANZANIA

NOTE: This questionnaire will be administered in a digitalized form on a tablet computer.

1. DATE: _____ / _____ / ________
2. Name of Interviewer: ____
3. Health facility: ________________________________
4. Unique identification number (IDNO): ___/_____(Site, serial number (e.g. 21/001)
5. GPS coordinates of household: LATITUDE __ __. __ __ __ __ LONGITUDE __ __. __ __ __ __
6. Name of participant: ____
7. Age: ___ years
8. Birth date: ___/___/____
9. Sex: ☐Male ☐Female
10. Mobile number: ____
11. Marital status: ☐ Single ☐ Cohabiting ☐ Married ☐ Separated ☐ Widow

12. Education level: ☐ No education ☐ Primary ☐ Secondary (I-IV) ☐ Colleges (certificate-Diploma) ☐ Graduate/Equivalent and above

13. Main occupation: ☐ Employee ☐ Farmer ☐ Livestock Keeper ☐ Fisherman ☐ other, specify:__________________
14. Body weight (Kg): _________. ____Kg
15. Height (cm): _______________cm

MEDICAL HISTORY

16. Type of COVID-19 vaccine
17. Number of doses administered
18. Interval between vaccine doses
19. Time since last vaccination
20. Previous SARS-CoV-2 infection
21. Co-morbid conditions: Do you currently have any of the following (tick)?

a) ☐ Organ transplant recipient
b) ☐ Diabetes (Type I or II)
c) ☐ Heart disease or heart problems
d) ☐ Hypertension (high blood pressure)
e) ☐ Overweight
f) ☐ A recent stroke
g) ☐ Kidney disease
h) ☐ Liver disease
i) ☐ Anaemia
j) ☐ Asthma
k) ☐ Other lung condition such as COPD, bronchitis or emphysema
l) ☐ Cancer
m) ☐ Condition affecting the brain and nerves (e.g. Dementia, Parkinson's, Multiple Sclerosis)
n) ☐ A weakened immune system/reduced ability to deal with infections (as a result of a disease or treatment)

o) ☐Depression

p) ☐Anxiety

q) ☐Psychiatric disorder

If yes, to: a, b, c, k, l, m, q; Please tell us the type

____

If yes to n; Please tell us why your immune system is weakened________________________________

22. For each of the following questions please consider your usual situation and respond (1=Yes, 2= No)
    a) In general, do you have health problems that require you to limit your activities?
    b) Do you need someone to help you on a regular basis?
    c) In general, do you have any health problems that require you to stay at home?
    d) If you need help, can you count on someone close to you?
    e) Do you regularly use a stick, walker or wheelchair to move about?

23. Do you currently take any regular medication? (1=Yes; 2=No)

If yes, what is it for?

____

____

History of COVID-19 and Testing

24. Have you ever suspected yourself to have COVID-19? (1=Yes, 2=No)

    If yes, when was that? (month/year _____/______

25. Have you ever had a test to see if you have or have had COVID-19? Tick all that apply:

    ☐No (Go to Qn 28)

    ☐Yes, because I had symptoms

    ☐Yes, because I have been in contact with someone who had COVID-19

☐Yes, because of my job

☐Yes, for another reason, please describe

…………………………………………………………

26. What kind of test have you had (tick all that apply)?

☐A swab test (swab taken from the throat or nose or saliva) which tests for active infection, including PCR tests.

☐An antibody test (this usually involves a drop of blood taken from your finger) which tests for past infection, including tests with a lateral flow device.

☐Other, please describe ________________________________

☐Don’t know

Was the test result positive? (1= Yes, 2=No,3=Don’t know)

When was the test done? DD/MM/YY ____/_______

27. Were you hospitalized for treatment of your COVID-19 disease? (1=Yes; 2=No, 8=Don’t know, 9=Prefer not to answer)

28. Have you had the flu/SARS vaccine? (1= Yes, 2=No)

If yes, when was that (month/year) ________/_______

BLOOD SAMPLE COLLECTION AND PROCESSING

Blood sample collected (1= yes, 2=no)

Date of sample collection……………………

Sample accepted (1= yes, 2=no)
